# Dual-specificity Phosphatase 9 protects against Cardiac Hypertrophy by targeting ASK1

**DOI:** 10.7150/ijbs.57130

**Published:** 2021-05-27

**Authors:** Lang Jiang, Lingyun Ren, Xin Guo, Jing Zhao, Hao Zhang, Shanshan Chen, Sheng Le, Hao Liu, Ping Ye, Manhua Chen, Jiahong Xia

**Affiliations:** 1 Department of Cardiovascular Surgery, Union Hospital, Tongji Medical College, Huazhong University of Science and Technology, Wuhan, China.; 2 Department of Anesthesiology, The Central Hospital of Wuhan, Tongji Medical College, Huazhong University of Science and Technology.; 3 Department of Cardiology, The Central Hospital of Wuhan, Tongji Medical College, Huazhong University of Science and Technology, Wuhan, China.

## Abstract

The functions of dual-specificity phosphatase 9 (DUSP9) in hepatic steatosis and metabolic disturbance during nonalcoholic fatty liver disease were discussed in our prior study. However, its roles in the pathophysiology of pressure overload-induced cardiac hypertrophy remain to be illustrated. This study attempted to uncover the potential contributions and underpinning mechanisms of DUSP9 in cardiac hypertrophy. Utilizing the gain-and-loss-of-functional approaches of DUSP9 the cardiac phenotypes arising from the pathological, echocardiographic, and molecular analysis were quantified. The results showed increased levels of DUSP9 in hypertrophic mice heart and angiotensin II treated cardiomyocytes. In accordance with the results of cellular hypertrophy in response to angiotensin II, cardiac hypertrophy exaggeration, fibrosis, and malfunction triggered by pressure overload was evident in the case of cardiac-specific conditional knockout of DUSP9. In contrast, transgenic mice hearts with DUSP9 overexpression portrayed restoration of the hypertrophic phenotypes. Further explorations of molecular mechanisms indicated the direct interaction of DUSP9 with ASK1, which further repressed p38 and JNK signaling pathways. Moreover, blocking ASK1 with ASK1-specific inhibitor compensated the pro-hypertrophic effects induced by DUSP9 deficiency in cardiomyocytes. The main findings of this study suggest the potential of DUSP9 in alleviating cardiac hypertrophy at least partially by repressing ASK1, thereby looks promising as a prospective target against cardiac hypertrophy.

## Introduction

Biomechanical stimuli like pathological mechanical pressure overload often indulge in cardiac hypertrophy, which is an important predisposing factor of heart failure 1-3. Although it initiates as a compensatory process to maintain output, sustained cardiac hypertrophy is attributed to the onset of maladaptation and pathological remodeling, resulting in structural, functional, and electrophysiological alternations 1, 4, 5. The progression of this cardiac abnormality under hypertrophic stresses involves the crosstalk between multitudinous signaling transduction cascades, including MAPKs, PI3K/AKT, and PKA 5-8. Thus, a better understanding of the pathophysiology and molecular mechanism may elicit possible strategies to combat pressure overload-induced cardiac hypertrophy and further progression to heart failure.

Dual-specificity phosphatase 9 (DUSP9), which has an alternative name as MAP kinase phosphatase-4 (MKP-4), acts as a well-identified dephosphorylated mediator 9. Through heralding its substrates on serine/threonine and tyrosine residues, DUSP9 is a key player in a wide range of intercellular pathways and pathological processes 9, 10. For instance, by inhibiting the canonical JNK phosphorylation, DUSP9 contributes to the fine-tuning of JNK, finally amending glucose intolerance and repressing hepatic steatosis in stress-induced insulin resistance 9. Recently, we have revealed the role of DUSP9 in binding to and dephosphorylating the apoptosis signal-regulating kinase 1 (ASK1), thereby switching off p38 and JNK-axis in the development of hepatic steatosis 11. In fact, DUSP9 has been found to illustrate a substrate preference for MAPK pathway, in particular ERK1/2, JNK, and p38 12, 13, all of which are associated with the development of cardiac hypertrophy 14.

Nonetheless, the role of DUSP9 in pressure overload-induced cardiac hypertrophy and heart failure has yet to be elucidated. However, our study witnessed marked up-regulation of DUSP9 in heart samples obtained from animals after the transverse aortic constriction (TAC) surgery and cultured cardiomyocytes suffering from angiotensin II (Ang II) challenge. Moreover, the utilization of gain- and loss-of-function strategies revealed increased susceptibility of cardiac-specific DUSP9-knockout mice to pressure overload-caused cardiac hypertrophy and malfunction, while such phenotypes were successfully restored by cardiac DUSP9 overexpression in transgenic mice. The molecular mechanism revealed direct physical interaction and dephosphorylation of ASK1 by DUSP9, which, in turn, inactivated its downstream p38/JNK signaling pathway. Moreover, the adverse consequences of DUSP9 absence could be rescued by abolishing ASK1 in response to cardiac hypertrophy after Ang II stimuli. Based on the above data, our findings substantiated the role of DUSP9 as a novel anti-hypertrophic mediator through targeting ASK1.

## Materials and methods

### Animals

All animals were maintained and bred in the Division of Laboratory Animal Resources at Tongji Medical College. All animal care and related experiments outlined in this study were carried out in accordance with the Guidelines for the Care and Use of Laboratory Animals drafted by US National Institutes of Health (NIH Publication, 8^th^ Edition, 2011). The Institutional Animal Care and Use Committee from both Tongji Medical College and Huazhong University of Science and Technology approved the animal protocols followed in this study.

### Generation of cardiac-specific DUSP9 knockout mice

DUSP9-floxed mice model was generated as reported in the previous study 15. The DUSP9 ^fl^°^x/fl^°^x^ (DUSP9-Flox) mice were mated with a-MHC-MerCreMer transgenic mice (Jackson Laboratory, 005650) to produce DUSP9^fl^°^x/fl^°^x^/MEM-Cre mice. The cardiomyocytes-specific conditional DUSP9 knockout (DUSP9-CKO) mice were induced by intraperitoneal injection of DUSP9 ^fl^°^x/fl^°^x^/MEM-Cre mice (at six weeks of age) with tamoxifen (Sigma-Aldrich, T-5648) (25 mg/kg per day) for five days. The DUSP9 ^fl^°^x/fl^°^x^/MEM-Cre mice (DFMC) without tamoxifen injection were considered as control mice.

### Cardiac-specific DUSP9 transgenic mice generation

Cardiac-specific overexpression of DUSP9 transgenic mice was established as detailed previously 15. In brief, the full-length mouse DUSP9 cDNA sequence was cloned into a backbone vector with a mouse a-MHC promoter. Then, the a-MHC-DUSP9 vector was linearized prior to the pronuclear microinjections into fertilized C57BL/6 embryos. The tail DNA samples were obtained to identify the founder transgenic mice using primers: sense 5'-CATAGAAGCCTAGCCCACACCA-3'; anti-sense 5'-TGAAGCTGGTTTCACACAGG-3', yielding a 709 bp product.

### Transverse Aortic Constriction (TAC) Surgery

As described previously, the TAC procedure or sham operation was executed 8-10 weeks old adult male mice 16. In brief, intraperitoneal injection of sodium pentobarbital was used to anesthetize the mouse, and the aortic arch was visualized from the right side of clavicle by opening the skin in the middle of chest. Then the aortic arch was fixed using a 26-G needle with 7-0 silk suture. The sham group mouse was performed with identical procedures without tied ligature in the same location. The self-regulating heating pad was used to maintain the body temperature of mice at about 37.0 °C during the process of surgery. After the surgery, mice were kept under observation in a 25.0 °C heated cage until recovery.

### Echocardiographic estimations

Inhalation of 1.5~2% isoflurane by mice on a 37 °C temperature-controlled warming pad was applied to achieve anesthetization. Then, a MyLabGamma ultrasound system equipped with an 18-MHz linear ultrasound transducer was utilized to estimate the cardiac structure and function. The heart rates of mice were monitored continuously, and the echocardiographic examination could be conducted when the heart rates remain around 420 bpm.

### Histological analysis

The hearts were rapidly harvested and soaked in 10% potassium chloride solution to preserve the heart at diastole, fixed in 10% formalin, further dehydrated, and embedded in paraffin, which were transversely cut into 5-µm sections. These heart sections were proceeded with HE (Hematoxylin and Eosin) and PSR (Picrosirius red) staining. Afterward, the sections were studied under light microscopy (Nikon Corporation, Japan). The cross-sectional area of cardiomyocyte and the degree of collagen deposition were examined by Image-Pro Plus 6.0 system with captured images.

### Neonatal rat ventricular myocytes (NRVM) isolation and culture

NRVM were detached from ventricles of 1day old neonatal Sprague-Dawley rats. Ventricles were cut into pieces and digested with collagenase and trypsin. The cell suspension was collected and centrifuged to collect the cell pellets. To remove fibroblasts, cells were seeded and maintained routine incubation condition for 2 hr, and then the non-adherent CMs were collected, re-suspended and seeded for culture while the adherent fibroblasts were disposed.

The cells undergone starvation under serum-free medium condition for 12 h to synchronize before treatment with Ang II (1 µM) or PBS or ASK1 inhibitor GS-4997(iASK1 80 µM, 1148428-04-3, Selleck) or DMSO for another 48 h.

### Immunofluorescence staining and double immunofluorescence assay

Cardiomyocytes on glass coverslips were fixed with room temperature 4% formaldehyde for 15 min, PBS wash for three times, and permeabilized by 0.2% Triton X-100. The cardiomyocytes were then stained with a primary antibody against α-actinin (Sigma, A7811, 1:100 dilution). 4',6-Diamidino-2-phenylindole (DAPI, Invitrogen, S36939) was applied to stain cell nucleus. The quantification of cell surface area was acquired through Image-Pro Plus 6.0 system.

The localization and interaction between DUSP9 and ASK1 were measured by double immunofluorescent analysis, as described previously 17.

### Co-immunoprecipitation (Co-IP)

HEK293T cell line was used for indicated expression vector transfection for 24 h. Co-immunoprecipitation was performed as described previously.

### Quantitative RT-PCR

In brief, total RNA was extracted using TRIzol (Invitrogen) according to manufacturer's instructions. Two microgram of mRNA was then reverse-transcribed into cDNA by exploiting a cDNA synthesis kit (Fermentas). RT-PCR was performed using SYBR Green/Fluorescein qPCR Master Mix kit (Fermentas) on the ABI Prism 7,500 platform.

### Western blot and antibodies

Western blot was carried out as described previously. Primary antibodies against DUSP9 (Proteintech, 10826-1-AP, 1:1000); p-ASK1 (CST, 3765, 1:1000); ASK1 (ABclonal, A6274, 1:1000); p-ERK (CST, 4370, 1:1000); ERK (CST, 4695, 1:1000); p-JNK (CST, 4668, 1:1000); JNK (CST, 9252, 1:1000); p-p38 (CST, 4511, 1:1000); p38 (CST, 8690, 1:1000); GAPDH (Proteintech, 60004-1-Ig, 1:5000); Flag (MBL, M185-3LL, 1:2000); HA (MBL, M180-3, 1:2000).

### Statistical analysis

Data were analyzed with SPSS (Statistical Package for Social Sciences) Statistics 21.0 software. Comparisons between two groups were computed using a 2-tailed Student's t-test. One-way analysis of variance (ANOVA) with the least significant difference (data meeting equal variance assumption) test or Tamhane's T2 was performed for data comparing more than two groups. The nonparametric analysis was used to scrutinize the sample size groups (n<4) and non-normally distributed data. *P*<0.05 was regarded as statistical significant.

## Results

### DUSP9 expression was increased in hypertrophic hearts and cardiomyocytes

**Figure 1:**
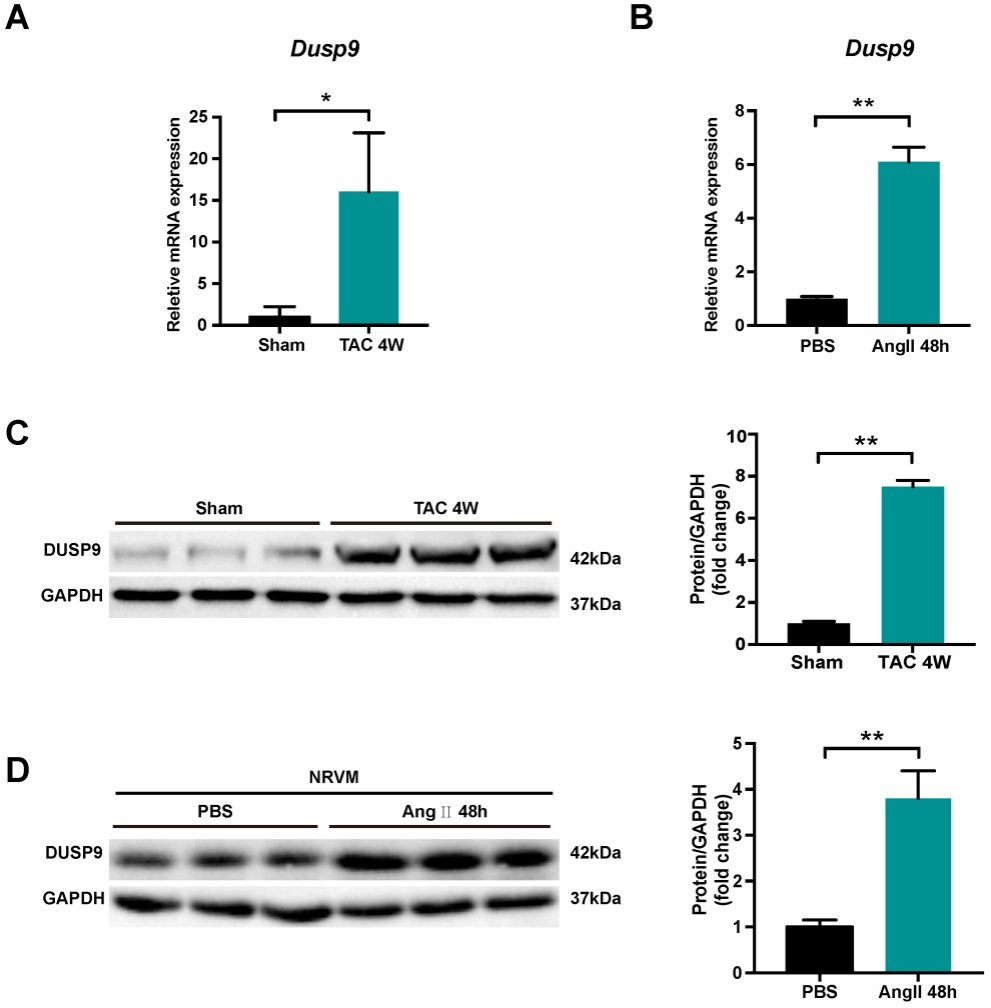
**DUSP9 expression was elevated in hypertrophic hearts and NRVM. (A)** The mRNA level of DUSP9 in heart tissue after TAC or sham procedures at specified time point (n = 3 per group). **(B)** The mRNA level of DUSP9 in NRVM treated with PBS or angiotensin II (Ang II, 1 µM) for 48 h. **(C)** Western blotting assay and DUSP9 protein level quantification in the mice heart after four weeks of TAC or sham procedures (n = 3 per group). **(D)** Western blotting, and quantification of DUSP9 protein level in NRVM treated with PBS or angiotensin II (Ang II, 1 µM) for 48 h. Data are presented as the mean ±SD. *P<0.05, **P<0.01; NRVM refers to neonatal rat ventricular myocytes.

DUSP9 expression was investigated to unravel its potential role in cardiac hypertrophy and heart failure development. Transverse aortic constriction (TAC) surgery was employed to induce pressure overload-induced cardiac hypertrophy in mice. Both mRNA and protein levels of DUSP9 were up-regulated in the murine heart undergoing TAC surgery for four weeks, as compared to the sham-operated group (Figure 1A, 1B). In comparison to control group, the escalation of DUPS9 mRNA and protein levels were observed in NRVM subjected to angiotensin II for 48 h (Figure 1C, 1D). Collectively, this evidence implicated a probable contribution of DUSP9 in the pathological mechanism of cardiac hypertrophy.

### Cardiac DUSP9 deficiency aggravated TAC-induced cardiac hypertrophy

**Figure 2:**
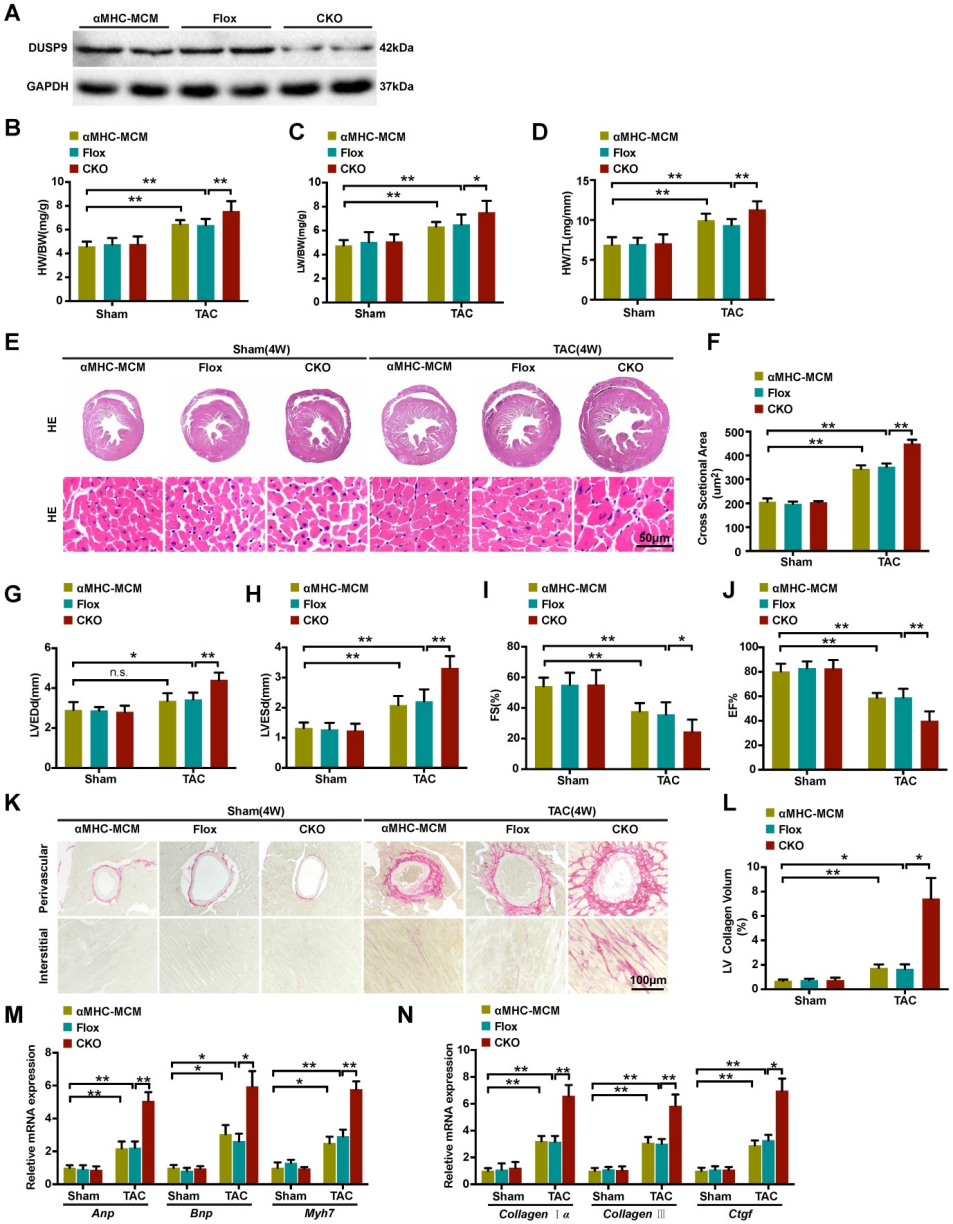
** Cardiac DUSP9 deficiency exacerbated TAC-induced cardiac hypertrophy. (A)** Representative western blot results to validate DUSP9 expression in **α**MHC-MCM, DUSP9-Flox and DUSP9-CKO mice (n = 6 per experimental group). **(B-D)** Statistical data of the ratios of Heart weight to body weight (HW/BW,** B**), lung weight to body weight (LW/BW, **C**) and heart weight to tibia length (HW/TL, **D**) in different genotypical mice (αMHC-MCM, DUSP9-Flox and DUSP9-CKO) at four weeks after sham or TAC procedures (n = 10 mice per group) **(E, F)** Histological analysis of gross morphology and left ventricular muscle of demonstrated groups four weeks after TAC surgery or sham procedures (n = 6 mice per group; scale bar, 50 µm). **(G-J)** Echocardiographic assessment of left ventricular end-diastolic diameter (LVEDd, **G**), left ventricular end-systolic diameter (LVESd, **H**), left ventricular fractional shortening (FS%, **I**) and left ventricular ejection fraction (EF%, **J**) in the demonstrated groups at four weeks after either sham or TAC procedures (n = 10 mice per group). **(K, L)** Representative imaging of PSR staining of perivascular and myocardial interstitial sections of the hearts from the demonstrated groups (n = 6 mice per group; scale bar, 100 µm) **(M, N)** Quantification results exhibiting the mRNA levels of hypertrophic biomarkers (*Anp*, *Bnp*, *Myh7*) and fibrotic markers (*collagen Iα*, *collagen III*, *Ctgf* ) in the indicated groups (n = 4 per group). Results are presented as mean ±SD. *P<0.05, **P<0.01.

We continue to explore the functional influence of DUSP9 in cardiac hypertrophy *in vivo*. The cardiomyocytes-specific conditional DUSP9 knockout (DUSP9-CKO) mice were developed with the tamoxifen-inducible α-MHC-Cre system. Western blotting assay confirmed the absence of DUSP9 in the heart of DUSP9-CKO mice (Figure 2A). Notably, no apparent abnormalities of cardiac phenotypes at baseline were witnessed in the DUSP9-CKO mice as compared to the control group. At the time of four weeks post TAC surgery, the hypertrophic parameters (HW/BW, LW/BW, and HW/TL) were enhanced in DUSP9-CKO mice compared with controls (Figure 2B-2D). Moreover, this finding was further validated by the HE analysis, which demonstrated thickened ventricular wall and enlarged cardiomyocyte cross-sectional area of the DUSP9-CKO mice than control mice after TAC surgery (Figure 2E, 2F). Relative to the control mice, reduced EF% and FS% accompanied by the elevations of LVEDd and LVESd in DUSP9-CKO mice also indicated obvious deterioration of cardiac function after TAC treatment (Figure 2G-2J). Moreover, cardiac fibrosis is a canonical pathological characteristic of cardiac hypertrophy. PSR staining of DUSP9-CKO mice also exhibited prominent features of cardiac fibrosis after TAC treatment in both perivascular and interstitial fibrosis with regards to the control group (Figure 2K, 2L). Furthermore, in concordance with these morphological and functional changes, higher mRNA levels of hypertrophic markers, including *Anp*, *Bnp*, *Myh7* (Figure 2M), together with fibrotic markers, including *collagen Iα*, *collagen III*, *Ctgf* (Figure 2N), were revealed in the hearts of DUSP9-CKO mice comparing to controls post TAC surgery. Collectively, these data indicated that the progression of pressure overload-induced cardiac hypertrophy and heart failure were aggravated in light of DUSP9 absence.

### Cardiac-specific DUSP9 overexpression attenuated TAC-induced cardiac hypertrophy

**Figure 3:**
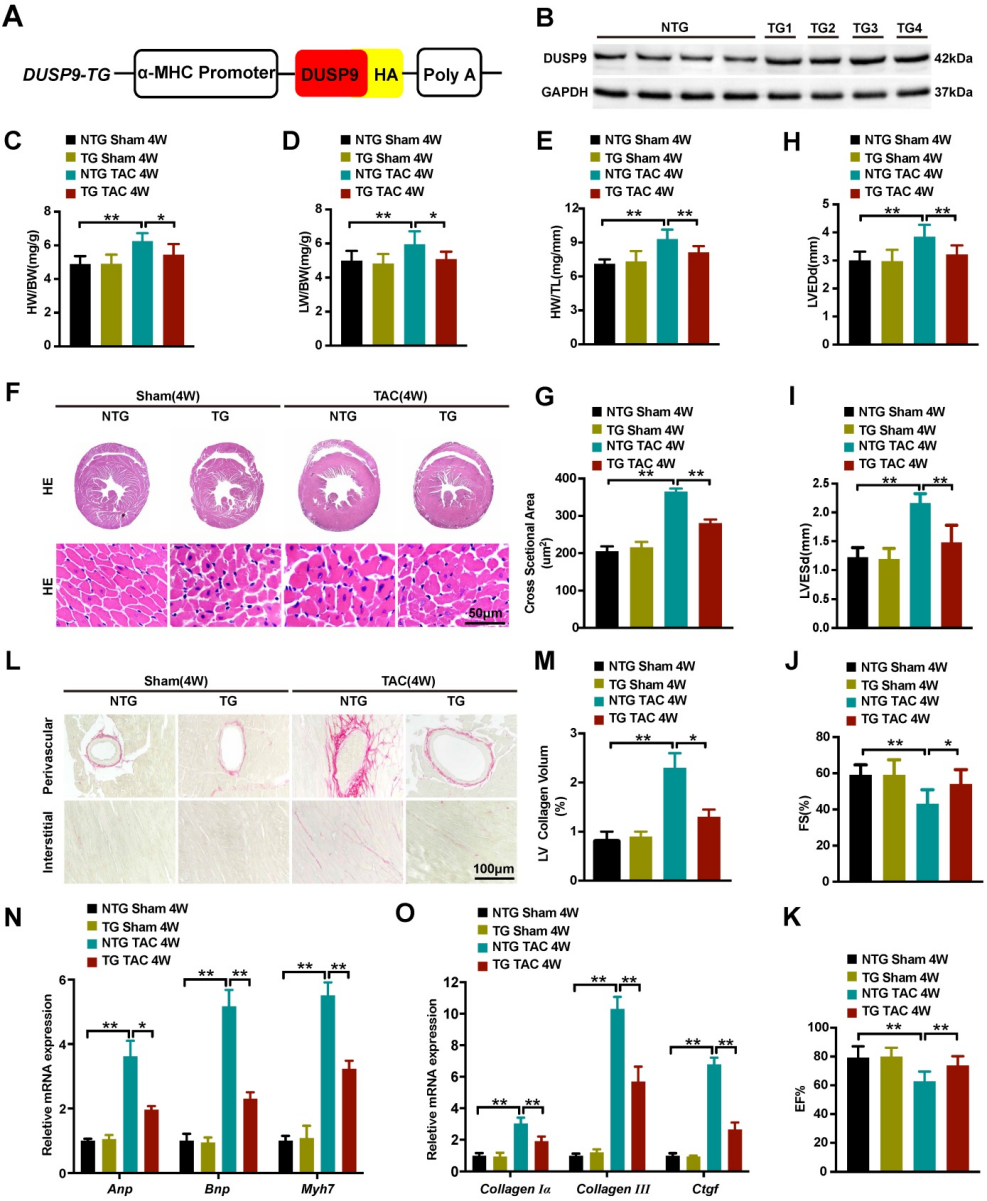
**Cardiac-specific DUSP9 overexpression attenuated TAC-induced cardiac hypertrophy. (A)** Schematic graphic illustrating the establishment of cardiac-specific DUSP9 transgenic mouse strains. **(B)** Cardiac DUSP9 expression in transgenic mice and non-transgenic littermates (n = 4 per group). **(C-E)** Statistical data of the ratios of HW/BW** (C),** LW/BW (**D),** HW/TL **(E),** in non-transgenic and transgenic mice at four weeks after sham or TAC surgery (n = 10 mice per group) **(F, G)** Histological images of the gross morphology and left ventricular muscle of heart stained with HE from the demonstrated groups four weeks after TAC surgery or sham procedures (n = 6 mice per group; scale bar, 50 µm) **(H-K)** Parameters of the echocardiographic assessment for LVEDd (**H**), LVESd (**I**), left ventricular FS% (**J**), and left ventricular EF% (**K**), in the demonstrated groups (n = 10 mice per group). **(L, M)** Representative imaging of PSR staining of perivascular and myocardial interstitial sections of the hearts from the demonstrated groups (n = 6 mice per group; scale bar, 100 µm) **(N, O)** The mRNA levels of hypertrophic biomarkers (*Anp*, *Bnp*, *Myh7*) and fibrotic markers (*collagen I*, *collagen III*, *Ctgf*) in the demonstrated groups at four weeks after TAC surgery or sham procedures (n = 4 per group). Data are presented as the mean ±SD. *P<0.05, **P<0.01.

Cardiomyocyte-specific DUSP9 transgenic (DUSP9-TG) mice were developed to assist in elucidating the ameliorative effect of DUSP9 in *in vivo* cardiac hypertrophy development. Here, we established four independent DUSP9-TG mouse lines, which were then confirmed by western blotting (Figure 3A, 3B). As expected, the cardiac function, histology, and morphology under the baseline displayed no apparent anomalisms in the case of cardiac-specific DUSP9-TG mice. However, four weeks after TAC treatment, the expression of DUSP9 in DUSP9-TG mice heart was significantly increased relative to the sham group (Figure S1) and significant alleviation of cardiac hypertrophy (characterized by the repression of the ratios of HW/BW, LW/BW, and HW/TL) was witnessed in DUSP9-TG mice in comparison to NTG controls (Figure 3C-3E). Dramatic amelioration of the TAC-induced heart enlargement attributed to DUSP9 overexpression was also prominent from the histological observations (Figure 3F, 3G). Furthermore, echocardiography manifested a markedly improved cardiac chamber and LV function in DUSP9-TG mice, post-TAC surgery, relative to the NTG group (Figure 3H-3K). The diminution in the volume of collagens in both perivascular and interstitial areas was evident from the PSR staining (Figure 3L, 3M). Moreover, the inductions of hypertrophic markers (*Anp*, *Bnp*, *Myh7*) (Figure 3N), together with the fibrotic markers (*collagen Iα*, *collagen III*, *Ctgf*) (Figure 3O) on transcription levels in response to TAC challenge were considerably attenuated in the heart of DUSP9-TG mice comparing to NTG controls. These outcomes substantiated the fact that cardiac-specific DUSP9 overexpression was successful in combating cardiac hypertrophy and heart failure* in vivo* after overload pressure induction.

### DUSP9 repressed Ang II-prompted hypertrophy of cardiomyocyte *in vitro*

**Figure 4:**
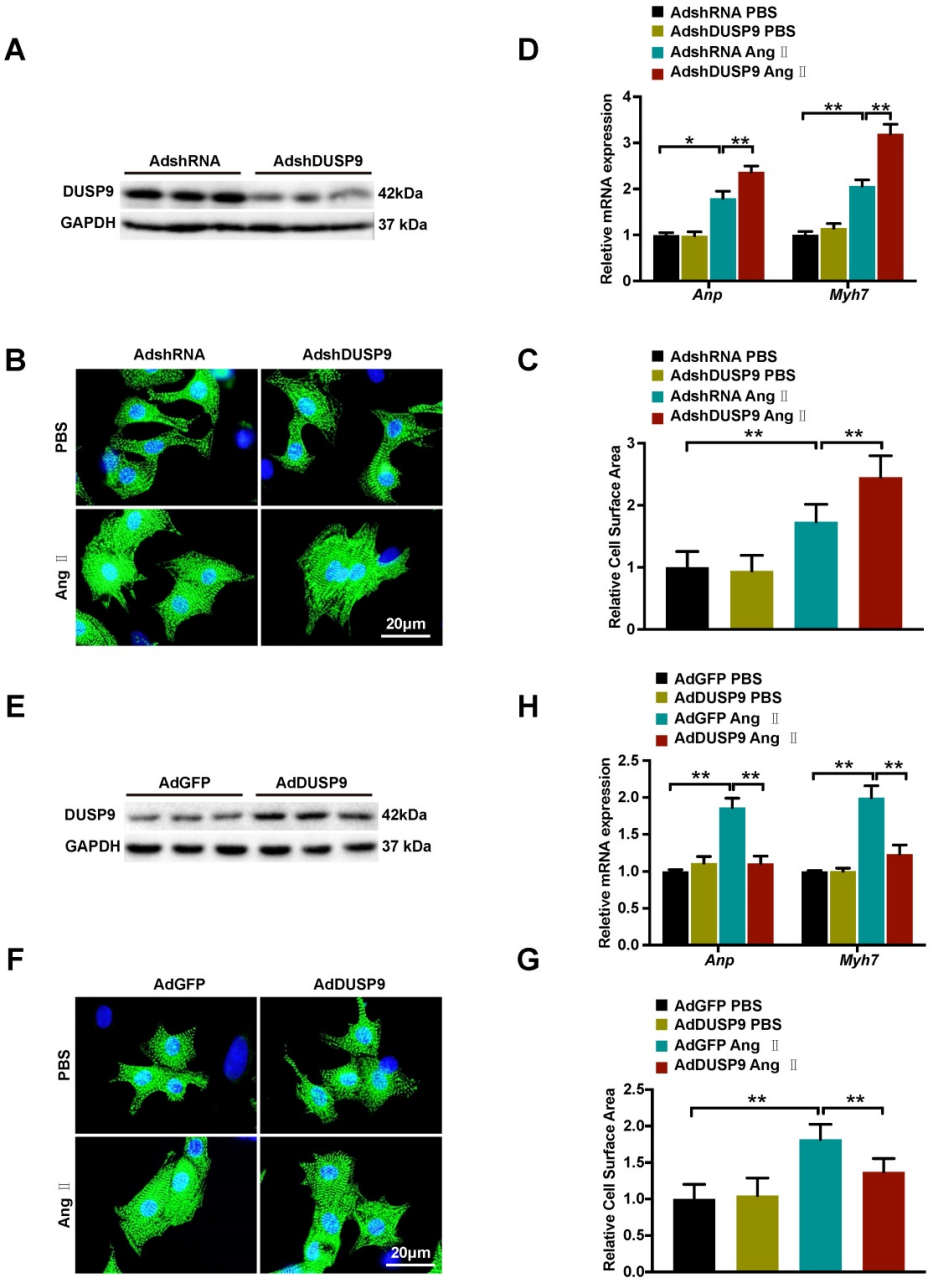
** DUSP9 repressed Ang** II**-induced NRVM hypertrophy *in vitro*. (A)** Respective western blots showing DUSP9 expression in NRVM infected with adenoviral vectors with shDUSP9 or controls **(B, C)** Representative immunofluorescence images of NRVM stained with α-actinin (green) and DAPI (blue) after infection by adenovirus of vectors with shDUSP9 or controls followed with a 48h-challenge of PBS or Ang II (1 µM). **(D)** The mRNA level of hypertrophic marker genes (*Anp* and *Myh7*) in NRVM after adenoviral vectors with shDUSP9 or controls infections undergoing Ang II (1 µM) or PBS treatment for 48 h **(E)** Respective western blots showing DUSP9 expression in NRVM infected with adenoviral vectors with Flag-DUSP9 or controls. **(F, J)** Representative immunofluorescence images of NRVM stained with α-actinin (green) and DAPI (blue) after the infections with adenoviral vectors with Flag-DUSP9 or controls followed by 48 h of PBS or Ang II (1 µM) challenge. **(H)** The mRNA levels of hypertrophic biomarkers (*Anp* and *Myh7*) in NRVM infected with adenoviral vectors with Flag-DUSP9 or controls undergoing Ang II (1 µM) or PBS for 48 h. Data are presented as the mean ±SD. *P<0.05, **P<0.01; NRVM refers to neonatal rat ventricular myocytes.

After demonstration of DUSP9 deficiency worsened while overexpression ameliorated cardiac hypertrophy *in vivo*, the gain- and loss-of-function experimentation were executed subsequently to determine the roles of DUSP9 on NRVM in response to cardiac hypertrophy *in vitro*. DUSP9 protein level was decreased by adenoviral vectors with shDUSP9 (Figure 4A) and increased by adenoviral vectors with Flag-DUSP9 in NRVM (Figure 4E). The cells were then subjected to Ang II or PBS treatment separately for 48 h, serving as treatment and control group, respectively. With Ang II stimulation, the adenoviral vectors with shDUSP9 transfected NRVM depicted marked augmentation of the cell surface area relative to the control groups (Figure 4B, 4C), whereas, in case of the adenoviral vectors with Flag-DUSP9 treated cells, it was notably limited in comparison to the control groups (Figure 4F, 4G). Hypertrophic markers, including *Anp* and *Myh7*, were detected after either Ang II or PBS treatment at mRNA level. Similar to the variation tendency of cell size, DUSP9 knockdown in NRVM with Ang II treatment resulted in a significant increase in these hypertrophic markers (Figure 4D), whereas DUSP9 overexpression was effective in reducing these hypertrophic parameters (Figure 4H). Thus, these findings distinctly verified that DUSP9 negatively impacted the pathophysiological state of cardiac hypertrophy and heart failure *in vitro*.

### DUSP9 modulated cardiac hypertrophy via the ASK1-p38/JNK signaling pathway

**Figure 5:**
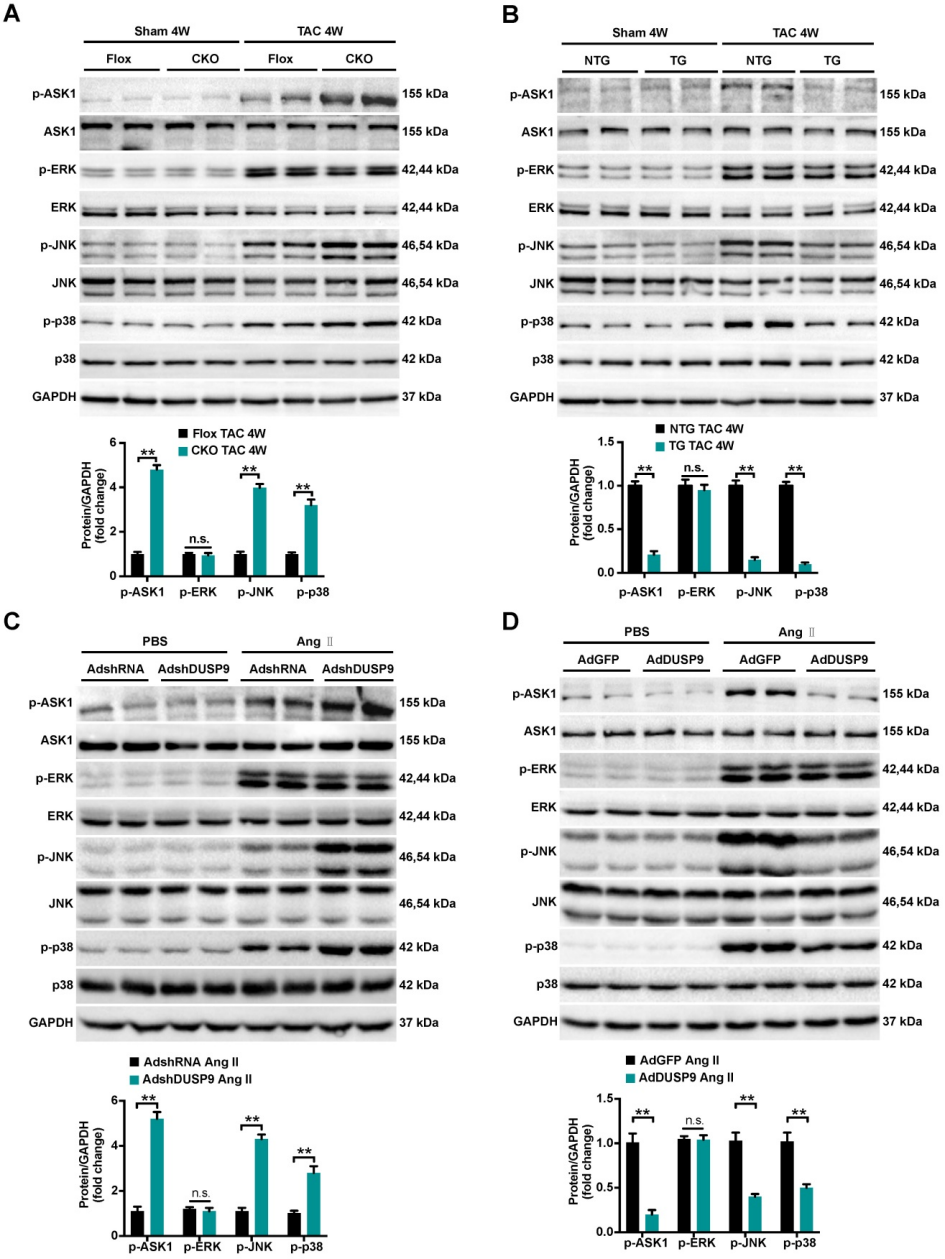
**DUSP9 modulated cardiac hypertrophy via the ASK1-p38/JNK1/2 signaling axis. (A, B)** Representative western blots and quantification of both phosphorylated and total protein levels of ASK1, p38, ERK1/2 and JNK in the hearts of control and DUSP9-CKO **(A)** or NTG and DUSP9-TG mice **(B)** four weeks after TAC treatment or sham (n = 4 mice per group) **(C, D)** Representative western blots and quantification of phosphorylated and total protein levels of ASK1, p38, ERK and JNK in NRVM with DUSP9 knockdown **(C)** or DUSP9 overexpression (**D**) after 48 h of PBS or Ang II (1 µM) challenge. Data are presented as the mean ±SD. *P<0.05, **P<0.01, n.s. means no significant difference; NRVM refers to neonatal rat ventricular myocytes.

To trace the potential molecular mechanisms behind this anti-hypertrophic action of DUSP9, we first highlighted the expression levels of MAPKs pathways, including p38, ERK1/2, and JNK, all are known to be important mediators responsible for the pathological cardiac hypertrophy 6, 8. As illuminated in Figure 5A and 5B, a dramatic increase in the phosphorylated expressions of ASK1, JNK, and p38 in mice heart were perceived four weeks after TAC surgery relative to the sham group. Notably, the pressure overload-induced p-ASK1, p-JNK, and p-p38 expressions were restored to basal levels after DUSP9 overexpression but were further facilitated in DUSP9-CKO mice subjected to TAC surgery. However, deficiency or forced expression of DUSP9 in mice heart failed to influence the protein expression of p-ERK1/2 in response to TAC surgery. In accordance with the results* in vivo*, DUSP9 knockdown further elevated Ang II-induced p-JNK and p-p38 levels without interfering with ERK expression. Meanwhile, DUSP9 overexpressed NRVM after Ang II stimulation witnessed a significant reduction in p-ASK1, p-p38, and p-JNK, whereas ERK1/2 remained unchanged (Figure 5C, 5D). The abovementioned results endorsed the anti-hypertrophic effect of DUSP9 was mediated via the ASK1-p38/JNK signaling pathway.

### DUSP9 regulates Ang II-induced cardiac hypertrophy through direct binding with ASK1

**Figure 6:**
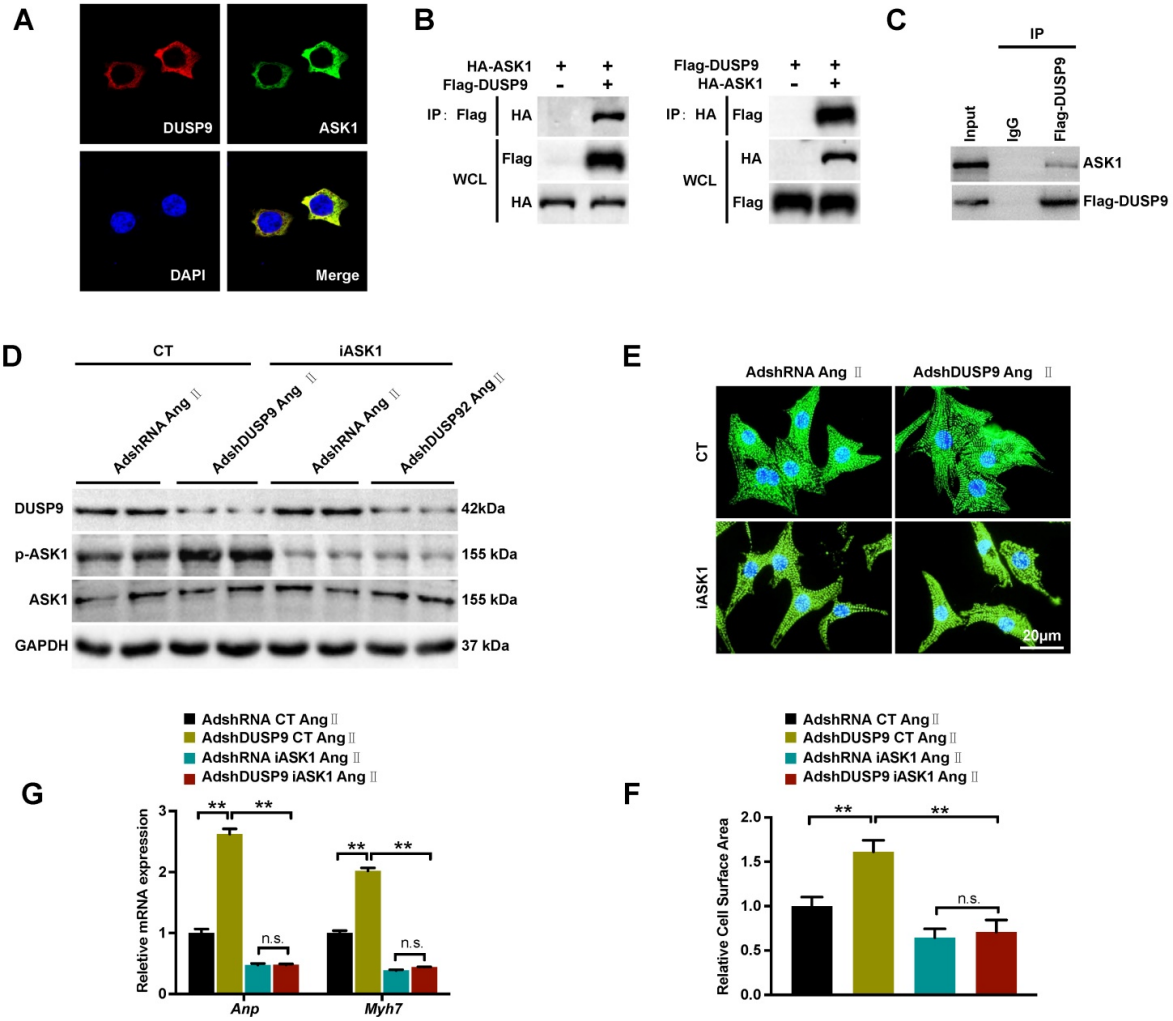
**DUSP9 regulated Ang II-induced cardiac hypertrophy by binding to ASK1. (A)** Immunofluorescence staining of DUSP9 (red), ASK1 (green) and nuclei (blue) in HEK293T cells. **(B)** Co-immunoprecipitation assay was performed in HEK293T cells that co-transfected with Flag-DUSP9 and HA-ASK1 to measure the interaction between DUSP9 and ASK1. **(C)** Co-immunoprecipitation assay in NRVM infected with Flag-DUSP9 to exam the interaction between DUSP9 and endogenous ASK1. **(D)** Western blots of DUSP9 and phosphorylated ASK1 protein level in DUSP9 knockdown NRVM treated with DMSO or iASK1 along with Ang II (1 µM) insult for 48 h. **(E, F)** Representative fluorescent images and quantification of cell surface area of DUSP9 knockdown NRVM with DMSO or iASK1 with Ang II (1 µM) stimulation for 48 h (Scale bar = 20 µm) **(G)** mRNA levels of hypertrophic biomarkers (*Anp* and *Myh7*) in DUSP9 knockdown NRVM undergoing Ang II (1 µM) treatment for 48 h along with DMSO or iASK1. Data are presented as the mean ±SD. *P<0.05, **P<0.01; NRVM refers to neonatal rat ventricular myocytes.

Based on the above results, DUSP9 repressed ASK1 phosphorylation, which in turn, reduced its downstream JNK and p38 proteins in response to cardiac hypertrophy. The double immunofluorescent analysis revealed a significant overlap of fluorescent signal for DUSP9 (red) and ASK1 (green), thereby confirming the exclusive co-localization of DUSP9 and ASK1 HEK293T cell cytoplasma (Figure 6A). The proposition that there might be a direct interaction between DUSP9 and ASK1 was, therefore, established by this finding. In order to address this question, co-IP was conducted using HEK293T cells, which have been co-transfected with Flag-DUSP9, and HA-ASK1 plasmids and in NRVM transfected with Flag-DUSP9. Physical interaction between DUAP9 and ASK1 was evident from the co-IP results (Figure 6B, 6C). Finally, to further detect whether the inactivation of ASK1 could reverse the abnormalities in the presence of DUSP9 deficiency, ASK1 activity was blocked with iASK1 in NRVM. Western blotting confirmed that Ang II-stimulated ASK1 phosphorylation was almost entirely abolished in iASK1-treated cells comparing to the respective control groups (Figure 6D). More importantly, the inactivation of ASK1 could significantly eliminate the DUAP9 knockdown-mediated increase in cardiomyocyte size (Figure 6E, 6F) along with elevated mRNA levels of hypertrophic indices (*Anp* and *Myh7*) (Figure 6G). To conclude, these findings suggested DUSP9 regulated Ang II-stimulated cardiac hypertrophy through directly binding to ASK1.

## Discussion

Our study was oriented to uncover previously unrecognized function of DUSP9 in pressure overload-induced cardiac hypertrophy in both* in vivo* and *in vitro* systems. Key findings discovered in this study were summarized as follows: (i) the levels of DUSP9 were strikingly increased in hypertrophic mice heart and Ang II-stimulated cardiomyocytes; (ii) cardiac hypertrophic response to TAC surgery was significantly intensified with the CKO-DUSP9 mice, whereas overexpression of DUSP9 in transgenic mice hearts attenuated hypertrophic alternations; (iii) the *in vitro* study also revealed the role of DUSP9 in repressing hypertrophy of Ang II-induced cardiomyocytes; (iv) mechanistic study delineated direct interaction and dephosphorylation of ASK1 by DUSP9, resulting in inactivation of MAPKs signaling. The adverse consequences of cardiac hypertrophy could be successfully recovered in DUSP9-knockout cardiomyocytes by blocking ASK1. Thus, our study, for the first time, provides evidence that establishes DUSP9 as an intrinsic negative mediator for pressure overload-caused cardiac hypertrophy.

The advancement of pressure overload-induced cardiac remodeling involves various crucial pathological modifications, including myocardial hypertrophy and fibrosis 4-6. Cardiac hypertrophy and fibrosis could affect each other in the progression of heart failure 18, 19. However, interpretation of the detailed mechanism of cardiac remodeling and heart failure remains a challenge 20, 21. The dual-specificity phosphatases (DUSPs) family has been known to be engaged in cardiac pathophysiology 22, 23; however, its effects differ with the stimulations and experimental conditions. Notable reduction of DUSP14 in pressure overload-induced cardiac hypertrophy has been reflected in the convincing data reported by Li and his colleagues 22. Contradictory to the result, Liu et al. found that Ang II-or phenylephrine (PE)-induced cardiomyocytes as well as hypertrophic hearts subjected to transverse aortic constriction (TAC) or myocardial infarction (MI) treatments portrayed increase in DUSP8 24. Interestingly, the present study documented significant up-regulation of DUSP9 in TAC-treated mice heart and Ang II-suffered cardiomyocytes during the pathological development of cardiac remodeling. Worsened hypertrophic response to chronic pressure overload was also observed owing to loss of cardiac-specific DUSP9, which could be successfully rescued by cardiac-specific DUSP9 overexpression. It is tempting to speculate that, with varied expression patterns, animal models, and genetic backgrounds, DUSPs could serve similar or different contributions in stress-induced cardiac remodeling.

DUSPs comprise a large group of phosphatases engaged in dephosphorylated both serine/threonine and tyrosine residues 25. Typically, DUSPs anchor MAPKs as substrates and modulate the triple kinase pathways of MAPKs signaling cascade. DUSPs determine the activity states of the upstream molecules of the MAPKs signaling pathway, including ASK1, TAK1, and MEK, which sequentially affect terminal MAPKs signaling molecules (ERK1/2, JNK, and p38) 26, 27. Thus, inhibition of the negative modulated molecular mechanisms resulting from DUSPs would result in uncontrolled canonical cascades that would detrimentally give rise to cardiac dysfunction, cardiac remodeling, together with heart failure during the pathological stimulus 24. Here, we identified that the cardiac DUSP9 overexpression in transgenic mice blunted the activation of ASK1 and its downstream p38/JNK in response to pressure overload or Ang II insult, whereas cardiac-specific DUSP9 knockout reversed the effects. The co-immunoprecipitation study that addressed how DUSP9 negatively modulates ASK1 activation, confirmed the physical interaction of DUSP9 with ASK1, suggesting that DUSP9 may shield the ASK1 protein to make it inaccessible to activation. Further experiments will be required to identify the interactive domains of these two proteins. Thus, we believe that DUSP9-mediated ASK1-p38/JNK axis may contribute to be a novel molecular mechanism in the onset of cardiac hypertrophy.

ASK1 belongs to the protein family of MAPK kinase (MAPKKK), with prominent expression in cardiomyocytes 28, 29. Once activated, ASK1 allows for phosphorylation and activations of MAPK kinase (MKK) (MKK3/6 and MKK4/7) and subsequently, transfers signals to the p38 and JNK, respectively 30. Recently, several studies have highlighted a critically detrimental role of ASK1 phosphorylation in regulating the pressure overload-induced cardiac hypertrophy and ASK1-mediated p38 and JNK signaling pathways can be nurtured as a potential target for alleviating cardiac remodeling 29, 30. Our present study is the first to confirm the direct binding of DUSP9 with ASK1 and acts as an important co-regulator for ASK1 dephosphorylation in pressure overload-and Ang II-induced cardiac remodeling. The present study also delineated that by directly interacting with ASK1, DUSP9 regulated p38/JNK but not ERK1/2 in pressure overload-induced cardiac hypertrophy. These findings were compliant with our previous report that documented the enhanced propensity of DUSP9 toward p38 and JNK without affecting ERK1/2 in non-cardiomyocytes. However, this was contradictory to the works of Kahn et al. that portrayed inhibition of ERK1/2 and JNK phosphorylation, and, to a lesser extent, p38, mediated by DUSP9 in anisomycin-induced insulin resistance 9. Variation of the effect of DUSP9 on the MAPKs signaling mediators depending on the cell and stimulus may partially justify this phenomenon.

In summary, DUSP9-ASK1-p38/JNK regulatory axis being an essential mechanism for cardiac hypertrophy is a novel target for preventing or delaying this pathological condition, which can be achieved by modulating this signaling axis.

## Supplementary Material

Supplementary figure.
