# Supplementary material for: Dual-specificity Phosphatase 9 protects against Cardiac Hypertrophy by targeting ASK1
Source: Int J Biol Sci. 2021 May 27;17(9):2193–204. doi: 10.7150/ijbs.57130 (PMC8241718; doi:10.7150/ijbs.57130)
Supplement: Supplementary file 1 — Supplementary figure. [file ijbsv17p2193s1.pdf]

Figure S1

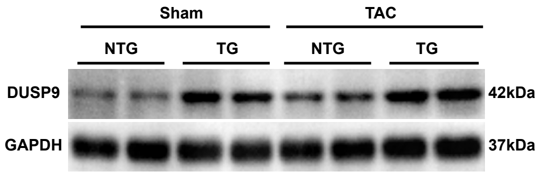

Supplementary Figure legends：

Figure S1. Representative western blot results to validate DUSP9 expression in non-transgenic and transgenic mice at four weeks after sham or TAC surgery (n = 4 mice per group)
